# Arginine Biosynthesis Mediates Wulingzhi Extract Resistance to Busulfan-Induced Male Reproductive Toxicity

**DOI:** 10.3390/ijms25126320

**Published:** 2024-06-07

**Authors:** Zifang Wu, Yuxuan Ma, Shaoxian Chen, Yuyan Liu, Xianglin Liu, Heran Cao, Tianqi Jin, Long Li, Mengqi Huang, Fangxia Yang, Wuzi Dong

**Affiliations:** 1 College of Animal Science and Technology, Northwest A&F University, Xianyang 712100, China; zifang_wu@126.com (Z.W.); mayuxuanrl@126.com (Y.M.); 15717875330@163.com (S.C.); 13683402668@163.com (Y.L.); caoheran@nwsuaf.edu.cn (H.C.); jintianqi2000@126.com (T.J.); 2021060188@nwafu.edu.cn (L.L.); h2036837547@163.com (M.H.); 2 College of Forestry, Northwest A&F University, Xianyang 712100, China; lxl6260686@163.com

**Keywords:** busulfan, Wulingzhi, testicular damage, oxidative stress, inflammation

## Abstract

Busulfan, an indispensable medicine in cancer treatment, can cause serious reproductive system damage to males as a side effect of its otherwise excellent therapeutic results. Its widespread use has also caused its accumulation in the environment and subsequent ecotoxicology effects. As a Chinese medicine, Wulingzhi (WLZ) has the effects of promoting blood circulation and improving female reproductive function. However, the potential effects of WLZ in male reproduction and in counteracting busulfan-induced testis damage, as well as its probable mechanisms, are still ambiguous. In this study, busulfan was introduced in a mouse model to evaluate its production of the testicular damage. The components of different WLZ extracts were compared using an untargeted metabolome to select extracts with greater efficacy, which were further confirmed in vivo. Here, we demonstrate abnormal spermatogenesis and low sperm quality in busulfan-injured testes. The WLZ extracts showed a strong potential to rehabilitate the male reproductive system; this effect was more prominent in room-temperature extracts. Additionally, both water and ethanol WLZ extracts at room temperature alleviated various busulfan-induced adverse effects. In particular, WLZ recovered spermatogenesis, re-activated arginine biosynthesis, and alleviated the increased oxidative stress and inflammation in the testis, ultimately reversing the busulfan-induced testicular injury. Collectively, these results suggest a promising approach to protecting the male reproductive system from busulfan-induced adverse side effects, as well as those of other similar anti-cancer drugs.

## 1. Introduction

In recent years, the prevalence of malignancies has been steadily increasing, and it is anticipated that the global burden of cancer in humans will continue to escalate in the forthcoming years [1], which has led to the continued increased use of anti-cancer drugs [2,3]. Moreover, over the past three decades, there has been extensive documentation indicating that pharmaceuticals typically persist in the environment following patient use, provoking significant ecotoxicological concerns across various environmental matrices [4]. Busulfan, functioning as an alkylating anti-cancer agent, has found broad applications in treating conditions such as lymphoma, chronic leukemia, and other malignancies [5]. Notably, it stands out as one of the few anti-cancer medications prescribed for children under the age of three [5,6]. Meanwhile, when considered among “hazardous drugs”, busulfan is also included in the National Institute for Occupational Safety and Health’s (NIOSH’s) group 1 list (NIOSH, 2020) [4]. Substantial evidence suggests that, while busulfan yields commendable outcomes, it also carries the potential to induce a range of toxic effects in mammals [6,7], especially in their reproductive systems [8,9]. Moreover, busulfan leads to the destruction of testicular germ cells, reduced sperm motility, higher rates of sperm abnormalities, and increased oligo-azoospermia, potentially resulting in temporary or permanent sterility [8,10]. Therefore, careful drug management is essential to lessen busulfan’s detrimental effects.

For thousands of years, traditional Chinese medicines have played crucial roles in treating various diseases. Wulingzhi (WLZ) is the dry extract of *Trogopterus xanthipes*, and as a traditional Chinese medicine, it enhances blood circulation and alleviates stasis [11]. In previous phytochemical studies, the main compounds of WLZ were found to include terpenoids, phenolic acids, flavones, sterols, and fatty acids [12,13]. Clinically, WLZ is often used to treat gynecological diseases such as amenorrhea, menstrual pain, and postpartum abdominal pain [12,14]. Recently, with progressing pharmacological research, more effects of WLZ have found applications. Previous research showed that WLZ can regulate the levels of antithrombin, inhibit platelet aggregation, enhance immunity, and act as an anti-inflammatory [13]. Another study revealed the functions of WLZ as an anti-atherosclerosis and an antifungal, as well as its ability to protect the gastric mucosa [15]. However, WLZ’s main function is related to support for female reproduction. The functions of WLZ in male reproduction remain ambiguous.

Interestingly, in terms of composition and bioactivities, WLZ presents potential for the treatment of reproductive injury induced by oxidation and inflammation. Therefore, in this study, we established a busulfan-induced testicular injury model to clarify the reproductive toxicity of busulfan. Meanwhile, different WLZ extracts were prepared, and we assessed the greater effects of WLZ extracts at room temperature on male reproduction using untargeted metabolomics. Consistently, in the in vivo models, both WLZ water and ethanol extracts presented an alleviating phenomenon in testes and sperm impaired by busulfan. Mechanistically, we unveiled that WLZ may alleviate busulfan-induced testicular injury through regulating oxidative stress and inflammation in the testis, which are both mediated by arginine biosynthesis signaling. In summary, this study revealed WLZ’s significant contribution to male reproduction and its potential mechanisms. Moreover, this study identified the best WLZ extraction method, suggesting that supplementation with WLZ extracts may be a promising strategy to restore fertility in male cancer patients undergoing busulfan treatment.

## 2. Results

### 2.1. Busulfan Exposure Caused Testicular Dysfunction and Abnormal Spermatogenesis in Mice

**Figure 1:**
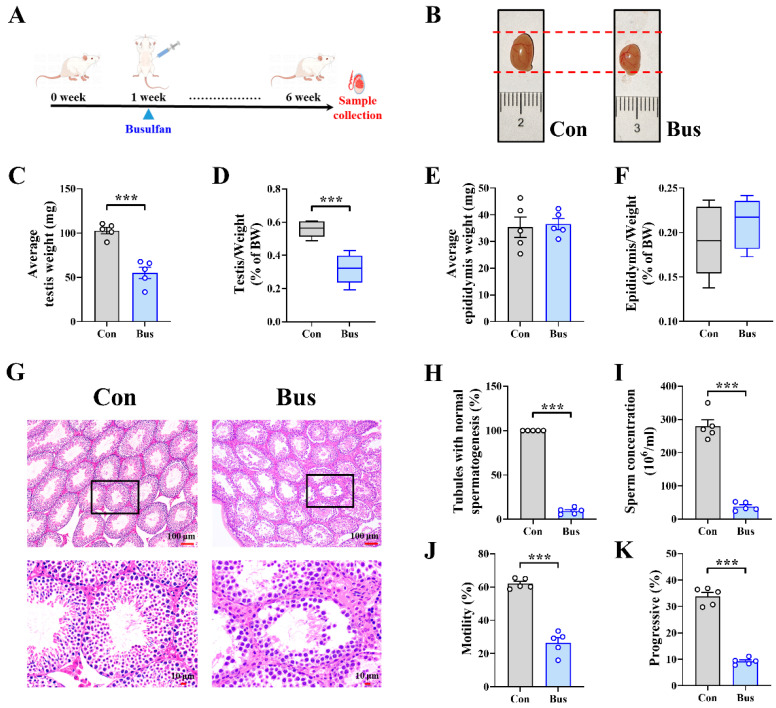
Effect of busulfan on testes of mice. (**A**) Schematic diagram of busulfan treatment during the test period. (**B**) Bright-field diagram of testicular size in control and busulfan injection groups. (**C**) Average testis weight. (**D**) Ratio of testis weight/body weight. (**E**) Average epididymis weight. (**F**) Ratio of epididymis weight/body weight. (**G**) Representative images of H&E staining in testis sections, with the 2nd row showing magnifications of the boxed regions in the 1st row; scale bar = 100 μm (1st row) and 10 μm (2nd row). (**H**) The percentage of normal spermatogenesis tubules in testicular samples. (**I**) Sperm concentration in epididymides. (**J**,**K**) Motility (**J**) and progressive motility (**K**) of sperm were assessed by computer-assisted semen analysis. *n* = 6 for each group. All data are presented as means ± SEM. Statistical significance was determined by the unpaired Student’s *t*-test. *** *p* < 0.001.

The busulfan infection model is shown in Figure 1A, although we found that the heart, liver, spleen, lung, and kidney organ indices did not show significant differences (Figure S1A–E). Still, the testicular weight and index were markedly reduced after busulfan treatment (*p* < 0.001) (Figure 1B–D). We also noticed that there was no difference in the epididymides’ weight and index (Figure 1E,F), which suggested that the damage caused by busulfan exposure mainly targets the testes.

To further characterize the testicular phenotype and analyze the effects of busulfan exposure on sperm, H&E staining was performed to detect pathological damage to the testes (Figure 1G). Compared with the Con group, the spermatogenic cells in seminiferous tubules were dramatically reduced and highly irregular, and those in the Bus group also displayed intraepithelial vacuolation. The proportion of tubules with normal spermatogenesis also supported more severe impaired spermatogenesis in the Bus group, where the number of spermatogenic cells was significantly reduced (90.69% down-regulation; *p* < 0.001) (Figure 1H). Moreover, sperm concentrations were significantly decreased in the Bus group (86.58% down-regulation; *p* < 0.001) (Figure 1I). In the busulfan group mice, significant sperm motility and progressive decreases were also found (*p* < 0.001) (Figure 1J,K). In addition, compared with the Con group, a series of sperm parameters were significantly lower in the Bus group mice (all *p* < 0.01) (Figure S1F–M). The above results all suggested that busulfan exposure is able to cause testicular dysfunction and abnormal spermatogenesis.

### 2.2. The Identification of Components in Different WLZ Extracts

**Figure 2:**
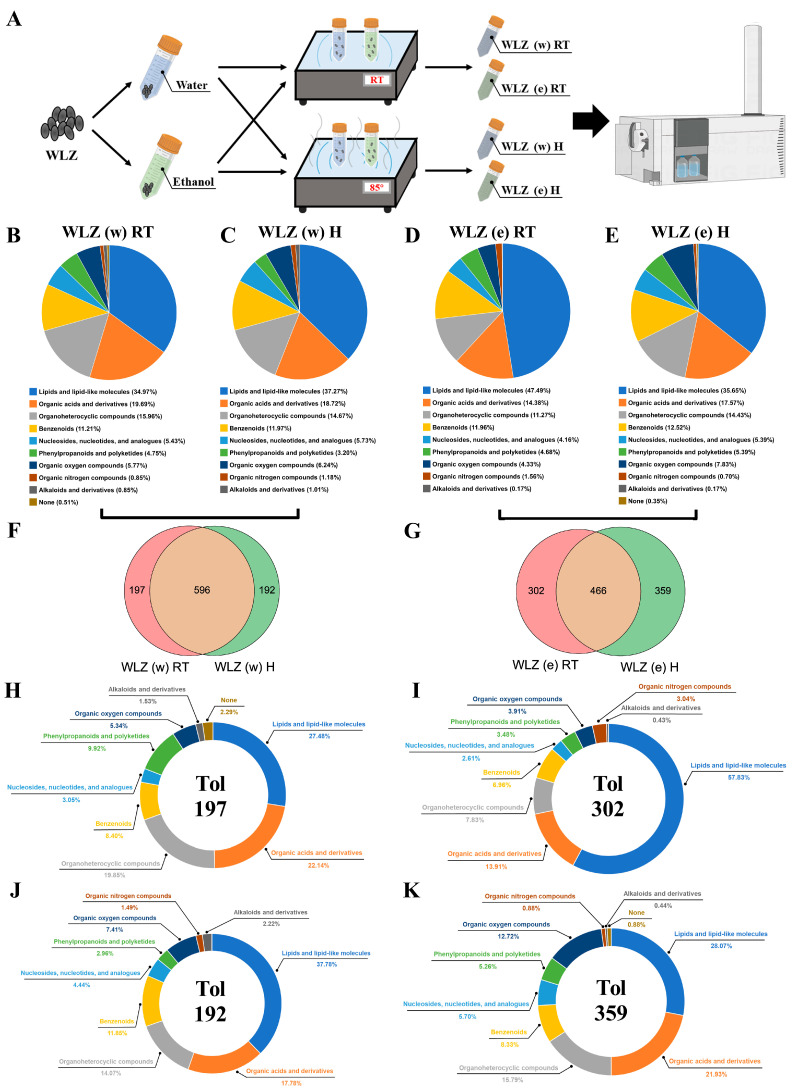
The composition changes in different WLZ extracts. (**A**) The untargeted metabolome profiles generated on different WLZ extract samples using an LC-MS (Liquid Chromatograph–Mass Spectrometer). WLZ (w) RT, the WLZ water extract at room temperature; WLZ (e) RT, the WLZ ethanol extract at room temperature; WLZ (w) H, the WLZ water extract at 85 °C; WLZ (e) H, the WLZ ethanol extract at 85 °C. (**B**–**E**) The components found in different WLZ extracts. (**F**,**G**) The Venn diagrams generated to describe the common and unique metabolites in the WLZ water extract (**F**) and WLZ ethanol extract (**G**) at different temperatures. (**H**–**K**) The components of the unique metabolites in WLZ (w) RT (**H**), WLZ (e) RT (**I**), WLZ (w) H (**J**), and WLZ (e) H (**K**). *n* = 3 for each group.

To address each WLZ extract’s components, untargeted metabolomics analyses of various WLZ extracts were prepared for each WLZ extract form [i.e., WLZ (w) RT, WLZ (e) RT, WLZ (w) H, and WLZ (e) H] (Figure 2A). Then, metabolites from the raw data of each group were identified through the mzCloud and mzVault libraries. As shown in Figure 2B–E, the identified metabolites in each group were analyzed based on their chemical classification; the metabolites were divided into nine main classes in the “Class Ⅰ” classification. In the WLZ water extract group, the heat treatment slightly reduced the proportions of “organoheterocyclic compounds” (15.96% to 14.67%) and “phenylpropanoids and polyketides” (4.75% to 3.20%), and slightly increased the proportion of “lipids and lipid-like molecules” (34.97% to 37.27%) (the changes in proportion were more than 1%). For the WLZ ethanol extract group, the “lipids and lipid-like molecules” were obviously enriched in the WLZ (e) RT group (47.49%). Still, this proportion was largely down-regulated after heating (35.65%).

To further evaluate the effect of the heated treatment on the composition of the WLZ extracts, we then compared the composition changes in the water and ethanol extracts of WLZ under heated and non-heated conditions, respectively. The Venn diagram showed a total of 596 metabolites in both the room-temperature and heated groups in WLZ water extracts. There were 197 and 192 unique metabolites found in the WLZ (w) RT and WLZ (w) H groups, respectively (Figure 2F). We further analyzed the composition of the unique metabolites in the WLZ (w) RT (Figure 2H) and WLZ (w) H groups (Figure 2J). Compared to the heated treatment, the room-temperature extract had higher proportions of “organic acids and derivatives” (22.14% vs. 17.78%), “organoheterocyclic compounds” (19.85% vs. 14.07%), and “phenylpropanoids and polyketides” (9.92% vs. 2.96%). At the same time, the proportions of “lipids and lipid-like molecules” (27.48% vs. 37.78%), “benzenoids” (8.40% vs. 11.85%), “organic oxygen compounds” (5.34% vs. 7.41%) were lower in the room-temperature extract. Interestingly, the “organic nitrogen compounds” were only detected in the heated-treatment extract (1.49%). For the WLZ ethanol extracts, as shown in Figure 2G, there were 466 metabolites in both WLZ (e) extract groups, and 302 and 359 unique metabolites were found in the WLZ (e) RT and WLZ (e) H groups, respectively. The proportions of “lipids and lipid-like molecules” (57.83% vs. 28.07%) and “organic nitrogen compounds” (3.04% vs. 0.88%) were higher in the room-temperature extract. At the same time, its proportions of “organic acids and derivatives” (13.91% vs. 21.93%); “organoheterocyclic compounds” (7.83% vs. 15.79%); “nucleosides, nucleotides, and analogues” (2.61% vs. 5.70%); “phenylpropanoids and polyketides” (3.48% vs. 5.26%); and “organic oxygen compounds” (3.91% vs. 12.72%) were lower (Figure 2I,K). These results indicate that different WLZ extract methods may have a significant influence on WLZ extract compositions, which may lead to changes in function.

### 2.3. The Untargeted Metabolomics Analyses of Different WLZ Extracts

**Figure 3:**
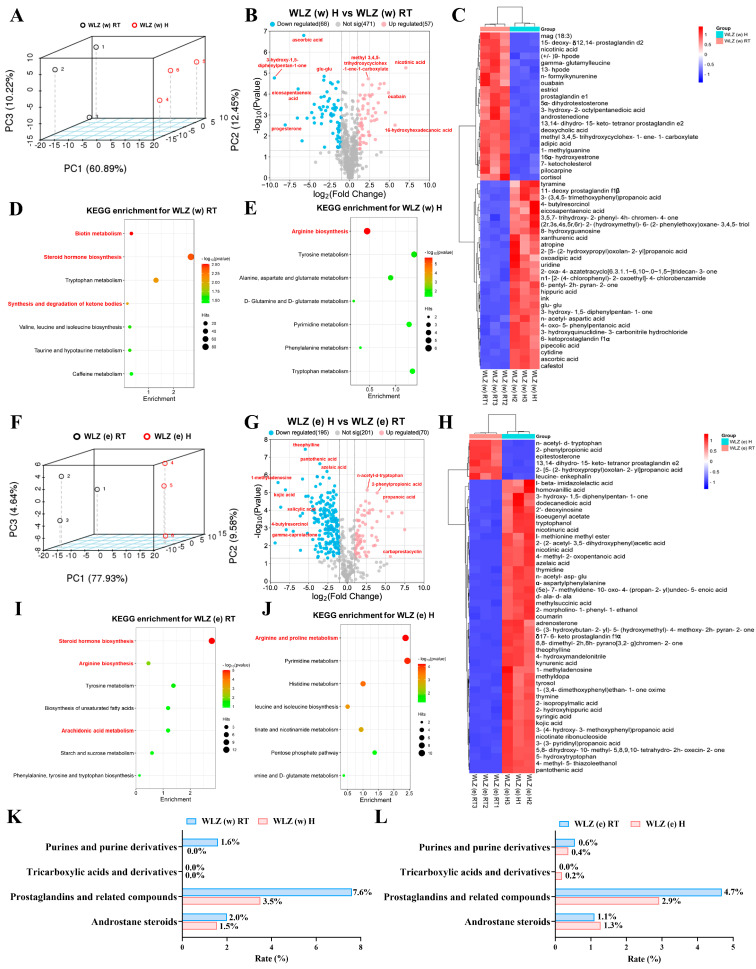
The metabolome changes in the WLZ water extract and WLZ ethanol extract at different temperatures. (**A**–**E**) The metabolomic changes in WLZ (w) RT and WLZ (w) H; (**A**) the 3D principal component analysis (PCA) score plot; (**B**) volcano plot showing the changes in metabolites; (**C**) heatmap of the top 50 differential metabolites; (**D**,**E**) the KEGG enrichment analysis of differential metabolites in WLZ (w) RT and WLZ (w) H. (**F**–**J**) The metabolomics changes in WLZ (e) RT and WLZ (e) H; (**F**) the 3D principal component analysis (PCA) score plot; (**G**) volcano plot showing the changes in metabolites; (**H**) heatmap of the top 50 differential metabolites; (**I**,**J**) the KEGG enrichment analysis of differential metabolites in WLZ (e) RT and WLZ (e) H. (**K**,**L**) The bar plot based on the differential metabolites which were enriched in the reproduction-related classes. *n* = 3 for each group.

In order to intensively investigate the potential functions of different WLZ extracts, we further conducted separate analyses on the WLZ water and ethanol extracts to determine the effects of heating on each of them. The 3D PCA score plot results showed that a distinct separation existed in the metabolites of the following groups: WLZ (w) RT vs. WLZ (w) H and WLZ (e) RT vs. WLZ (e) H (Figure 3A,F). Additionally, the PLS-DA model also effectively highlighted the distinction between the two paired groups (Figure S2A,E). The R^2^ and Q^2^ values revealed the good accuracy of the OPLS-DA models (Figure S2B,F). The data summary of the metabolites is presented in Figure 3B,G. Differential metabolites between groups were identified based on the criteria of FC > 2 and *p* < 0.05. Further analyses were conducted as detailed below.

WLZ water extracts, when compared to WLZ (w) H, as shown in Figure 3B, displayed 57 up-regulated and 68 down-regulated metabolites in WLZ (w) RT. The expression levels of the top 50 differentially expressed metabolites were analyzed and are presented in Figure 3C. Next, we grouped the above-mentioned up-regulated metabolites and the unique metabolites in WLZ (w) RT and compared its total up-regulated metabolites to WLZ (w) H (the metabolites in Figure 2H). We grouped the differential and unique metabolites in Figure 2J as the total down-regulated metabolites in the same way. Through pathway and KEGG enrichment analysis, these metabolites were mapped onto the specific pathways to elucidate the unique impacts of different WLZ extracts on metabolic pathways. The total up-regulated metabolites were mainly enriched in some reproduction-related pathways, such as “biotin metabolism”, “steroid hormone biosynthesis”, and the “synthesis and degradation of ketone bodies” (Figure 3D and Figure S2C). However, the total down-regulated metabolites were also enriched in a reproduction-related pathway (arginine biosynthesis). They were also enriched in some other pathways, such as that of “tyrosine metabolism” and “alanine, aspartate and glutamate metabolism” (Figure 3E and Figure S2D). For WLZ ethanol extracts, compared to WLZ (e) H, there were 70 up-regulated and 195 down-regulated metabolites in WLZ (e) RT (Figure 3G). The expression levels of the top 50 differentially expressed metabolites were analyzed and presented in Figure 3H. In the same way, these and the unique metabolites in Figure 2I,K were grouped into total up-regulated metabolites and total down-regulated metabolites. After pathway and KEGG enrichment analysis, the total up-regulated metabolites in WLZ ethanol extracts were also mainly enriched in several reproduction-related pathways (“steroid hormone biosynthesis”, “arginine biosynthesis”, and “arachidonic acid metabolism”) (Figure 3I and Figure S2G). Similarly, the total down-regulated metabolites were enriched in “arginine and proline metabolism” and other pathways (“pyrimidine metabolism”, “histidine metabolism”, and so on) (Figure 3J and Figure S2H).

The above-mentioned results suggest that the room-temperature extracts may have a better stimulating effect on the male reproductive function compared to the heated extracts. But considering that the heated extracts also experienced some reproduction-related pathway enrichment, we further targeted several metabolite classes, which were related to reproduction, to enrich differential and unique metabolites. As shown in Figure 3K, the WLZ (w) RT had more metabolites enriched in the reproduction-related classes, especially in the “purines and purine derivatives” (1.6% vs. 0.0%) and “prostaglandins and related compounds” (7.6% vs. 3.5%) pathways. Similar results were observed in WLZ ethanol extracts. WLZ (e) RT had more metabolites enriched in “prostaglandins and related compounds” (4.7% vs. 2.9%), although WLZ (e) H had a slight enrichment advantage in “tricarboxylic acids and derivatives” and “androstane steroids” (both 0.2% more than room-temperature extracts) (Figure 3L). Overall, the data further confirmed that the WLZ extracts at room temperature had more positive effects on male reproductive function.

### 2.4. The Metabolic Changes in WLZ (w) RT and WLZ (e) RT

**Figure 4:**
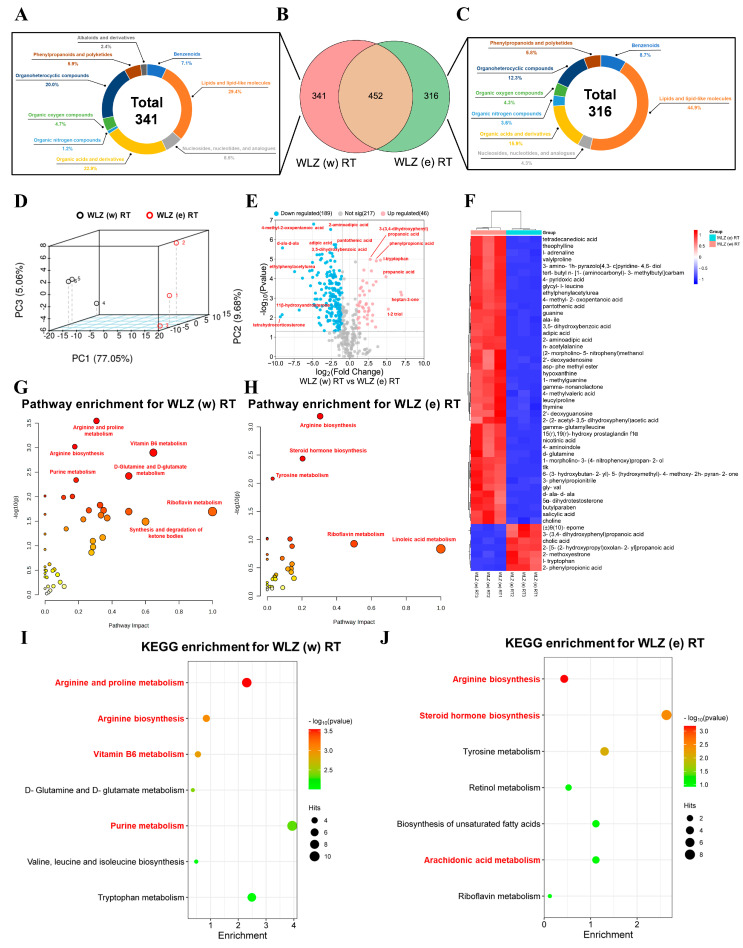
The metabolome changes in WLZ water and ethanol extracts at room temperature. (**A**–**C**) The Venn diagram was generated to describe the common and unique metabolites in the WLZ water extract and WLZ ethanol extract at room temperature, and the components of the unique metabolites. (**D**) The 3D principal component analysis (PCA) score plot. (**E**) Volcano plot showing the changes in metabolites. (**F**) Heatmap of the top 50 differential metabolites. (**G**,**H**) The pathway enrichment analysis of differential metabolites in WLZ (w) RT and WLZ (e) RT. The darker the color and larger the shape of the circle, the stronger the pathway influence. (**I**,**J**) The KEGG enrichment analysis of differential metabolites in WLZ (w) RT and WLZ (e) RT. *n* = 3 for each group.

As we observed the positive effects on reproduction from two room-temperature WLZ extracts, we next compared the metabolic characteristics between WLZ (w) RT and WLZ (e) RT in order to determine which extract enforced the greater potential impact on reproduction. As shown in Figure 4A–C, there were 452 metabolites in both WLZ (w) RT and WLZ (e) RT and 341 and 316 unique metabolites in WLZ (w) RT and WLZ (e) RT, respectively. Chemical classification analysis showed that the unique metabolites in WLZ (w) RT had greater proportions of “organic acids and derivatives” (22.9% vs. 15.9%) and “organoheterocyclic compounds” (20.0% vs. 12.3%). At the same time, the unique metabolites in WLZ (e) RT had a greater proportion of “lipids and lipid-like molecules” (44.9% vs. 29.4%). The 3D PCA analysis of the shared metabolites showed that a distinct separation existed in two groups (Figure 4D), and it screened out the differential metabolites using the standard as described previously. Moreover, compared to WLZ (w) RT, there were 46 metabolites up-regulated and 189 metabolites down-regulated in WLZ (e) RT (Figure 4E). The expression levels of the top 50 differentially expressed metabolites are analyzed and presented in Figure 3F. Finally, the pathway and KEGG enrichment analysis of unique metabolites is shown in Figure 4A, and the down-regulated metabolites showed that the metabolic pathway of WLZ (w) RT is mainly enriched in several reproduction-related pathways (“arginine and proline metabolism”, “arginine biosynthesis”, “vitamin B6 metabolism”, and “purine metabolism”). Interestingly, similar results were observed in WLZ (e) RT: “arginine biosynthesis”, “steroid hormone biosynthesis”, and “arachidonic acids metabolism” were mainly enriched in the differential and unique metabolites of WLZ (e) RT. In summary, the results above suggest that the main metabolic functions of the two WLZ extracts both pertain to male reproduction.

### 2.5. WLZ Extract Supplementation Alleviated Busulfan-Induced Testis Injury

**Figure 5:**
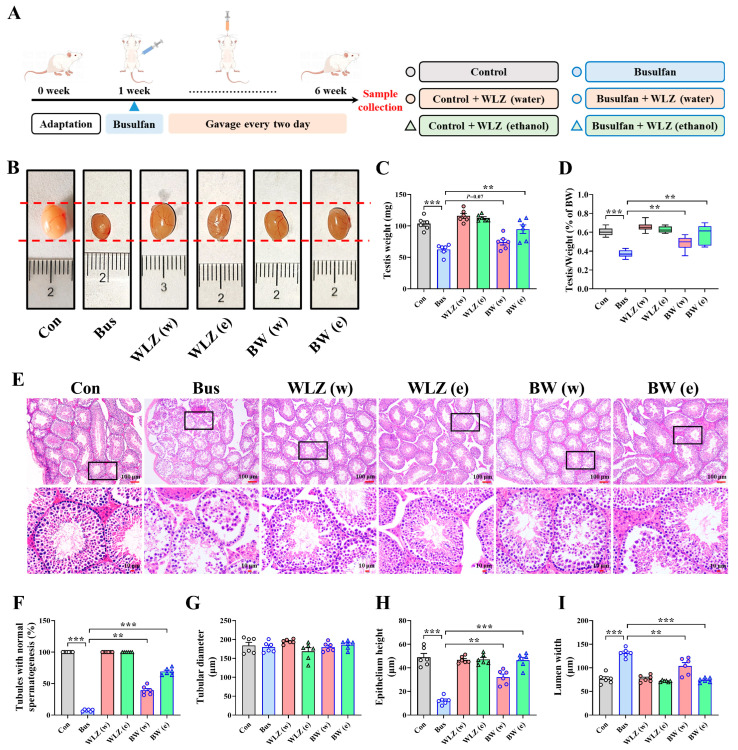
Effects of different WLZ extracts on testis injury caused by busulfan. (**A**) Schematic diagram of experiment. (**B**) Bright-field diagram of testicular size. (**C**) Average testis weight. (**D**) Ratio of testis weight/body weight. (**E**) Representative images of H&E staining in testis sections, with the 2nd row showing magnifications of the boxed regions in the 1st row; scale bar = 100 μm (1st row) and 10 μm (2nd row). (**F**) The percentage of normal spermatogenesis tubules in testicular samples. (**G**–**I**) The tubular diameter (**G**), epithelium height (**H**), and lumen width (**I**) of the testis. *n* = 6 for each group. All data are presented as means ± SEM. Statistical analysis was conducted using one-way ANOVA followed by Sidak’s multiple comparisons test. ** *p* < 0.01, *** *p* < 0.001.

After finding that WLZ room-temperature extracts had a positive potential function in male reproduction, our focus shifted towards investigating the potential of WLZ in alleviating testicular injury in vivo. The model shown in Figure 1 was used. Different WLZ extracts (Figure 4) were administered by gavage every two days after injection with busulfan or DMSO (Figure 5A). The results showed significant alleviation of testicular injury after WLZ water and ethanol extract treatment [BW (w) and BW (e)], including in testis weight (*p* = 0.07, *p* < 0.01) and testis index (both *p* < 0.01) (Figure 5B–D). However, the treatment did not influence the epididymis weight, epididymis index, or organ index of mice (Figure S3A–C). H&E staining was performed to further characterize the testis phenotype (Figure 5E). The results suggested that both the water and ethanol extracts could significantly recover the busulfan-decreased percentage of normal spermatogenesis tubules (*p* < 0.01, *p* < 0.001) (Figure 5F). However, the busulfan or WLZ treatment did not change the testis tubular diameter (Figure 5G). Still, busulfan significantly reduced the epithelium height (*p* < 0.001) and increased the lumen width of the testis (*p* < 0.001). In contrast, the WLZ treatment could recover these (Figure 5G–I). These results suggested that both WLZ water and ethanol extracts could significantly alleviate the busulfan-impaired testis function.

**Figure 6:**
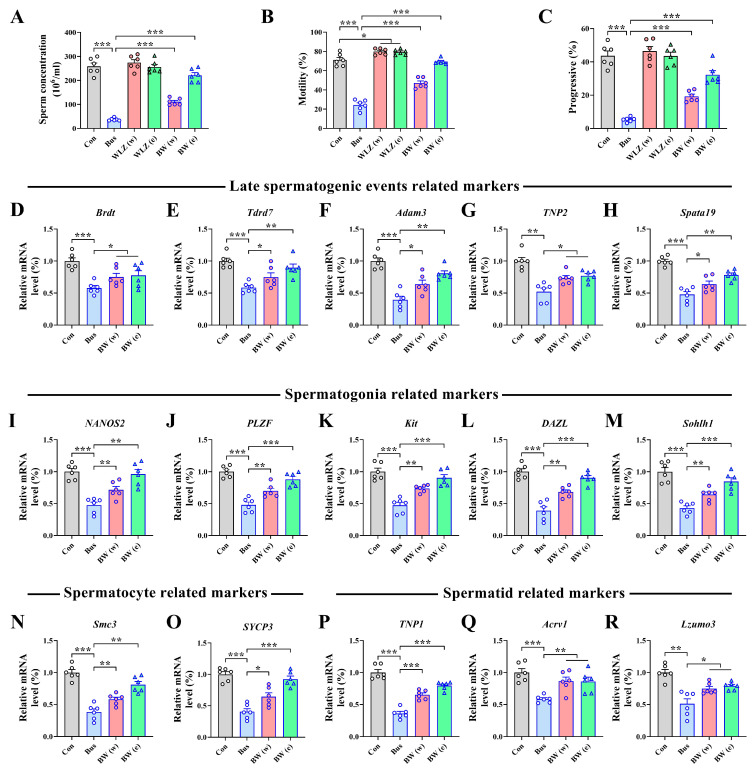
Changes in sperm parameters and marker genes involving spermatogenesis after WLZ treatment. (**A**) Statistical analysis of sperm concentration. (**B**,**C**) Motility and progressive motility of sperm. (**D**–**R**) Real-time PCR analysis of the mRNA expression of late-spermatogenic-event-related markers (**D**–**H**), spermatogonia-related markers (**I**–**M**), spermatocyte-related markers (**N**,**O**), and spermatid-related markers (**P**,**R**). *n* = 6 for each group. All data are presented as means ± SEM. Statistical analysis was performed using one-way ANOVA followed by Sidak’s multiple comparisons test. * *p* < 0.05, ** *p* < 0.01, *** *p* < 0.001.

Moreover, to further investigate changes in sperm quality and spermatogenesis signaling in the testes, we examined their sperm parameters and the expression of spermatogenesis-related markers. Specifically, we observed a decreased concentration (*p* < 0.001), motility (*p* < 0.001), and progressive motility (*p* < 0.001) of sperm in the busulfan treatment, which could all be reversed by WLZ treatment (all *p* < 0.05) (Figure 6A–C). The sperm parameter data also confirmed the above-mentioned observed results (Figure S3D–K). Since the WLZ treatment appeared to have no effect on the testes and sperm in the Con group [WLZ (w) and WLZ (e)], the additional study mainly focused on the BW (w) and BW (e) groups. For the spermatogenesis process, we measured the mRNA expression of a series of spermatogenesis-related genes. We observed that the expression of the mRNAs involved in late spermatogenic events was significantly decreased after busulfan treatment, including *Brdt* (*p* < 0.001) (bromodomain testis-specific factor), *Tdrd7* (*p* < 0.001) (Tudor domain-containing 7), *Adam3* (*p* < 0.001) (metallopeptidase domain 3), *TNP2* (*p* < 0.01) (transition protein 2), and *Spata19* (*p* < 0.001) (spermatogenesis-associated 19). However, the WLZ extract treatment improved these reduced genes (all *p* < 0.05) (Figure 6D–H). Similarly, we also observed that the WLZ extracts also recovered the mRNA expression of the markers of spermatogonia (marked by *NANOS2*, *PLZF*, *Kit*, *DAZL*, and *Sohlh1*), spermatocytes (marked by *Smc3* and *SYCP3*), and spermatids (marked by *TNP1*, *Acrv1*, and *Lzumo3*), which reduced after busulfan treatment (all *p* < 0.05) (Figure 6I–R). These results indicate that WLZ extracts safeguard spermatogenesis by enhancing the progression from spermatogonia to spermatocytes and ultimately to spermatids.

### 2.6. WLZ Extracts Alleviate the Oxidative Stress and Inflammation in Busulfan-Treated Testes via Arginine Biosynthesis

**Figure 7:**
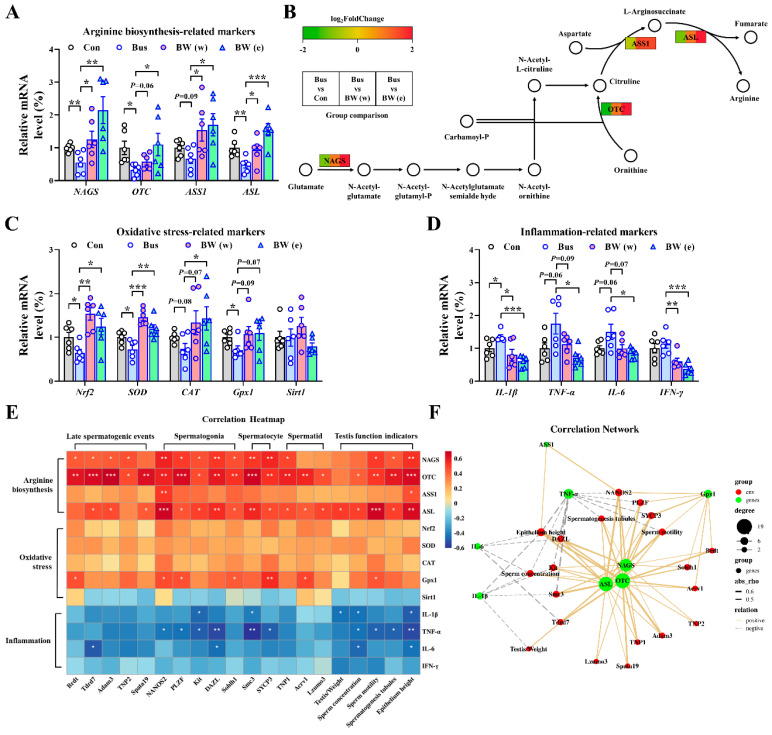
WLZ regulated arginine biosynthesis to affect the oxidative stress and inflammation in the testes. (**A**) Real-time PCR analysis of the mRNA expression of arginine biosynthesis-related markers. (**B**) Gene expression in arginine biosynthesis pathway. Green represents down-regulated genes, while red represents up-regulated genes in the Bus group. (**C**,**D**) Real-time PCR analysis of the mRNA expression of oxidative stress-related markers (**C**) and inflammation-related markers (**D**). (**E**) Sperman’s correlation between testis-related indicators and arginine-related indicators. (**F**) The correlation network of significant markers in (**E**). *n* = 6 for each group. All data are presented as means ± SEM. Statistical analysis was conducted using one-way ANOVA followed by Sidak’s multiple comparisons test. * *p* < 0.05, ** *p* < 0.01, *** *p* < 0.001.

**Figure 8:**
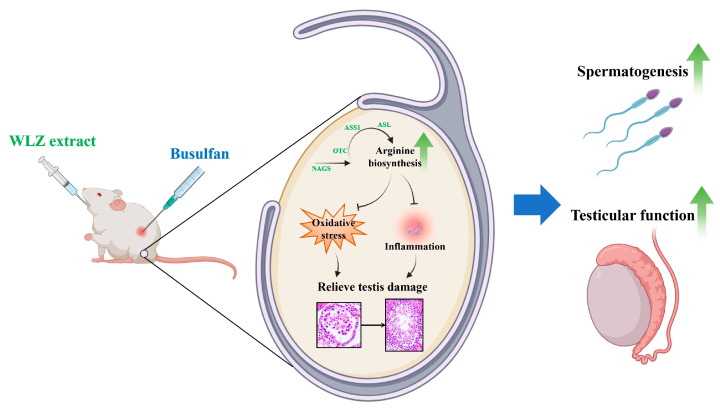
Schematic diagram for the hypothetical molecular mechanism of arginine biosynthesis-mediated WLZ resistance to busulfan-induced testicular dysfunction. Busulfan exposure led to impaired testicular function, mainly manifesting as oxidative stress and inflammation of the testes, abnormal spermatogenesis, and decreased sperm parameters, while WLZ supplementation induced arginine biosynthesis, which then relieved the increased oxidative stress and inflammation in the testes and finally rescued the spermatogenesis and testis function injured by busulfan.

In the previous results, we found that both the water and ethanol WLZ extracts can alleviate impaired testis function. Moreover, the pathway and KEGG enrichment showed that the arginine biosynthesis pathway was significantly enriched in the metabolites of both WLZ extracts. Thus, we further measured the expression of the key genes involved in arginine biosynthesis (map00220) (Figure 7A,B). The mRNA expression showed that busulfan reduced the mRNA expression of nearly all arginine biosynthesis-related genes, including *NAGS* (*p* < 0.01), *OTC* (*p* < 0.05), *ASS1* (*p* = 0.09), and *ASL* (*p* < 0.01). The WLZ extracts could re-increase the expression of arginine biosynthesis-related genes. As a functional amino acid, recent studies revealed that arginine plays an important role in regulating oxidative stress and inflammation [16,17,18]. Coincidentally, the spermatogenic cells were highly susceptible to oxidative insult, leading to a deleterious effect on spermatozoa [19,20], and the inflammation in the testis was also closely associated with germ cell aplasia [21,22]. Our findings showed that the restarted arginine biosynthesis in the testes displayed a strong correlation with the recovered spermatogenesis in the WLZ treatment. Thus, we further strove to clear up the possibility that WLZ extracts re-up-regulated arginine biosynthesis in the testes to alleviate the busulfan-induced testis function, which may be associated with oxidative stress and inflammation in the testis. To clarify this hypothesis, we determined the expression changes in the markers involved in oxidative stress and inflammation in the testes. As shown in Figure 7C,D, busulfan significantly down-regulated the mRNA levels of the oxidative stress-related genes *Nrf2*, *SOD*, and *Gpx1* (*p* < 0.05) and had a decreasing trend in *CAT* (*p* = 0.08). Both WLZ extracts had a significant or trending re-increase in the expression of these genes. Oppositely, busulfan up-regulated the mRNA expression of inflammation-related genes. At the same time, WLZ extracts reversed these phenomena. At last, we performed Spearman’s correlation analysis on the spermatogenesis process indicators and the expression of arginine signaling (Figure 7E). The correlation network based on these is shown in Figure 7F. The arginine biosynthesis key genes *NAGS*, *OTC*, and *ASL* were most positively correlated with the spermatogenesis process indicators, such as sperm motility; spermatogenesis tubules; and epithelium height, and the gene marker expressions of late spermatogenic events, spermatogonia, spermatocytes, and spermatids. Moreover, the anti-oxidative factor Gpx1 was positively correlated with most indicators of spermatogonia, spermatocytes, and testis function, while the inflammatory factors IL-1β, TNF-α, and IL-6 were negatively correlated with them. Collectively, these data further indicated that arginine biosynthesis may be involved in the recovery of WLZ-induced testicular injury, which may be mediated by the regulation of oxidative stress and inflammation in the testes, thus assisting in the recovery of spermatogenesis and testicular function (Figure 8).

## 3. Discussion

Over the past few decades, there has been increasing research focus on antineoplastic drugs in the environment and their potential ecotoxicological impacts. A previous study showed that the projection for the global human cancer burden is 28.4 million cases in 2040, a 47% rise from 2020 [1]. Therefore, the consumption of antineoplastic drugs is bound to increase, thereby increasing the risk of drug pollution. For example, as healthcare workers might be exposed to “dangerous” pharmaceuticals at many points during their daily practice, their offspring have shown an increased risk of fetal abnormalities and learning disabilities [23]. As a widely used alkylating anti-cancer agent, busulfan will adversely affect the male reproductive system, including by way of oligospermia or azoospermia, and may even lead to permanent male sterility [24,25]. In the present study, our major finding was the effectiveness of WLZ room-temperature extracts in alleviating busulfan-induced injury to the male reproductive system. This phenotype was probably mediated by the re-driven arginine biosynthesis signaling decreasing oxidative stress and inflammation in the testis. Collectively, we offer a new insight into a previously unrecognized effect of WLZ in promoting testicular function; this is, to the best of our knowledge, the first time a relationship between male fertility and WLZ has been described.

Due to not having specific target proteins, alkylating chemotherapy agents such as busulfan could induce widespread ROS-mediated oxidative damage, which is why they are always used in the treatment of middle and terminal cancers characterized by multiple gene mutations [26,27]. These factors also pose challenges in alleviating the adverse effects that these drugs produce. A previous study showed that the male sterility induced by busulfan in mice is very similar to that in humans [28]. In the current investigation, we comprehensively measured the busulfan-induced reproductive toxicity from spermatogenesis to sperm in multiple directions. Similar to previous studies, one dose of busulfan during youth could produce borderline azoospermia in mice during adulthood [29,30]. These appeared as significant developmental impairments from the testis to the sperm, a reduction in testis weight and index to almost half after busulfan treatment, nearly completely deteriorated spermatogenesis in the testis, and severe abnormal sperm parameters observed in the surviving sperm. Collectively, the consensus is that busulfan-induced testicular toxicity contributes significantly to male reproductive system impairment through disrupting spermatogenesis and sperm quality.

Previously, WLZ has displayed an effective curative effect for the treatment of gynecological diseases as well as immunity regulation and anti-inflammatory and anti-atherosclerosis properties [12,13,15]. However, owing to the complexity of its bioactive substances, the analysis of its components and processing methods to further explore the therapeutic potential of WLZ is essential. Considering the chemical components of WLZ, most of the main compounds of WLZ have shown significant potential antioxidant and anti-inflammatory effects, such as terpenoids, phenolic acids, and flavones [31,32,33]. Interestingly, these all implied the potential therapeutic ability of WLZ in testis damage induced by busulfan, which is mainly mediated by oxidative stress [34,35]. Here, we used an UPLC–Orbitrap–MS/MS method to evaluate the component changes in WLZ extracts at different temperatures (heated or room temperature) and with different solvents (water or ethanol). As expected, the different processing methods resulted in considerable variation in the WLZ extract composition, which is not unique in different preparation forms of the same drug [36,37]. Previous studies on the pharmacologically active components of Chinese medicine preparation mainly targeted analyses for the quantitation of a few marker components [37,38,39]. However, most of the preparations are mixtures containing hundreds of compounds or even more [40], and some markers may not even be the bioactive components of the medicine formula. Therefore, we used untargeted metabolomics to profile and compare various WLZ extracts with the aim of exploring their potential effects and molecular mechanisms. Ultimately, we hope to screen out the most promising WLZ extracts in the treatment of busulfan-induced testicular injury, which will be used for the subsequent animal tests.

In the extraction process, the solvent type and temperature are usually the two most influential factors affecting the quality and components of the extracts [41,42]. Similarly, in our results, although both the room-temperature and heated extracts affected reproductive-related metabolism, the room-temperature extracts contained significantly more metabolites related to reproduction, whether in the water or ethanol extracts. These metabolites included nicotinic acid [43], prostaglandin [44,45], dihydrotestosterone [46], cortisol [47], and epitestosterone [48,49], all of which play important roles in male reproduction. The enrichment analysis further confirmed the steroid hormones’ (such as dihydrotestosterone and cortisol) direct maintenance of male characteristics via the activation of the androgen receptor [50]. Arachidonic acid is also critical in male fertility and is also the main precursor for the synthesis of eicosanoids, including prostaglandins [51]. As a B-complex vitamin, biotin is considered to regulate immunological and inflammatory functions [52], and a previous study also reported that biotin supplementation could increase spermatogonia proliferation in the testes [53]. Ketone bodies could maintain the body’s redox homeostasis in response to environmental and metabolic challenges [54], and the therapy potential of the ketogenic diet for obesity also implies its alleviating effect on male reproduction [55]. Moreover, arginine is also known as a functional amino acid effect that promotes spermatogenesis and sperm quality [56]. Interestingly, in the additional analysis, both the room-temperature WLZ extracts (water and ethanol extracts) showed strong therapeutic potential in male fertility. The function pathways of WLZ water extracts tended to be enriched in pathways involving water-soluble metabolites such as arginine, vitamin B, and purine metabolism. On the contrary, the ethanol extracts of WLZ tended to be enriched in steroid hormone and arachidonic acid metabolism. However, we noticed that the arginine biosynthesis was also enriched in WLZ (e) RT, which may be due to the arginine-related metabolites being significantly enriched in water extracts. They are also slightly soluble in ethanol; thus, they could also be detectable in ethanol extracts. Collectively, all results implied the greater efficacy of room-temperature WLZ extracts on male reproductive function.

Our results demonstrated the busulfan-induced testicular toxicity and the potential effect of room-temperature WLZ extracts on male fertility. To clarify the role of WLZ extracts in busulfan-induced testis injury in vivo, busulfan-treated mice were gavaged with WLZ water or ethanol extracts. Consistent with our hypothesis, both the water and ethanol extract treatment effectively alleviated the impaired spermatogenesis and recovered the decreased concentration and motility of sperm. In addition, based on our data, the beneficial effects of WLZ were mainly due to the re-increased prominent genes affecting spermatogenesis. These were similar to previous studies [29,57]. The ROS-mediated oxidative damage was demonstrated to be the key mechanism of busulfan-induced damage to spermatogonia [58,59]. Coincidentally, arginine effectively regulated oxidative stress, improved immunity, and reduced inflammation [17,60]. The arginine biosynthesis pathway was also enriched in both two gavage extracts; thus, we proposed that WLZ alleviated the busulfan-induced testis damage through decreasing testicular oxidative stress and inflammation through arginine biosynthesis. The mRNA expressions of key markers of arginine biosynthesis, oxidative stress, and inflammation confirmed our hypothesis. As a previous study reported, *NAGS*, *OTC*, *ASS1*, and *ASL* were involved in the biosynthesis of arginine, starting with glutamine and ornithine [61]. In LPS-induced ovine intestinal epithelial cells, Gpx1 could be increased via arginine treatment to alleviate the oxidative damage caused by LPS [62]. Arginine could also alleviate the spleen inflammation of rats through regulating the transcription of inflammatory cytokine mRNA (including IL-1β and TGF-α) [63]. Our correlation analysis also revealed numerous strong correlations between the spermatogenesis process indicators and the arginine signaling expression, which further documented that the WLZ-activated arginine biosynthesis affected the anti-oxidant and anti-inflammatory functions in the testes, thereby improving testis health. Moreover, we observed interesting phenomena: the treatment with WLZ did not completely prevent busulfan-induced damage to the male reproductive system, and the ethanol extracts seemed to be more effective than the water extracts in mice. These results imply that a mixed extract of WLZ may be considered in our future research to investigate its underlying mechanism in the male reproductive system. Admittedly, this might provide a novel option for the development of drugs to reduce the adverse effects of busulfan.

## 4. Materials and Methods

This study was approved by the Animal Ethics Committee of Northwest A&F University (AAEWV-NWSUAF-DK2021050), and all the experiments were in accordance with the Guide for the Care and Use of Laboratory Animals at Northwest A&F University.

### 4.1. Preparation of Different WLZ Extracts

The WLZ was purchased from Jiuzhoutongsheng Agriculture and Animal Husbandry Technology Co., Ltd. (Shaanxi, China), which collected WLZ from Shangluo, Shaanxi, China (34.0897° N latitude and 110.1462° E longitude), and the company conducted the identification and quality control of the WLZ. The preparation of heated WLZ extracts was conducted in reference to our previous pre-experimental studies and several previous studies [64,65]. Briefly, 10 g of WLZ was weighed and homogenized with 100 mL sterile saline or ethanol absolute, followed by ultrasonic treatment in a water bath at 85 °C for 60 min twice. The samples were then filtered in 100 mL sterile saline or ethanol absolute filtrate. The water extract samples were stored at exactly −80 °C [WLZ (w) H], and the ethanol extract samples were dried in a vacuum concentrator at 37 °C and reconstituted in 100 mL sterile saline before being stored at −80 °C for further analysis [WLZ (e) H]. To ensure methodological consistency, the preparation of room-temperature WLZ extracts [WLZ (w) RT and WLZ (e) RT] was the same as for the heated WLZ extracts, except for the use of room-temperature water baths in the ultrasonic treatment.

### 4.2. Animals and Experimental Design

Healthy 4-week-old male ICR mice were procured from the animal center of the Fourth Military Medical University. They were then placed in a controlled environment (temperature ranging between 22 °C and 24 °C, a 12 h light/12 h dark cycle, and free access to food and water). Following a one-week adaptation period, the five-week-old mice were randomly assigned to two treatment groups (*n* = 6 mice for each group): the control group (Con) and the busulfan group (Bus). Mice in the Bus group were administered a single intraperitoneal injection of busulfan (40 mg/kg body weight) diluted in dimethyl sulfoxide (DMSO), while the Con-group mice were injected with normal DMSO. At 10 weeks old (5 weeks after injection), the mice were sacrificed. Testes, epididymides, and organ weight were recorded from the mice of all groups. The epididymides of the mice were used for the determination of sperm parameters, and the testes from the mice were fixed in 4% paraformaldehyde or stored at −80 °C for further analysis.

Further, to elucidate the impact of WLZ, mice were randomly distributed into 6 groups using identical methods (*n* = 6 mice for each group): mice in groups 1 and 2 were injected with normal DMSO or busulfan (40 mg/kg body weight) [Con and Bus]; mice in groups 3 and 4 were injected with normal DMSO and gavage with 200 μL normal temperature WLZ water or ethanol extract every 2 days for 5 weeks [WLZ (w) and WLZ (e)]; mice in groups 5 and 6 were injected with DMSO-diluted busulfan (40 mg/kg body weight), and the rest were treated in the same manner as groups 3 and 4 [BW (w) and BW (e)]. All mice were sacrificed 5 weeks after injection and analyzed as above.

### 4.3. Sperm Analysis

The fresh cauda epididymides of mice were dissected and sliced into pieces, then incubated in 1 mL of M2 medium (Sigma, M7167, St. Louis, MO, USA) at 37 °C in a 5% CO_2_ incubator for 10 min to cause sperm suspension. The suspended sperm was collected and analyzed using a computer-assisted sperm analyzer (CASA; HVIEW, Shenzhen, China) following the instruction protocol. The sperm parameters are detailed in a previous study [66], including motility, progressive motility, wobble, VCL (curvilinear velocity), VSL (straight-line velocity), VAP (average path velocity), ALH (amplitude of lateral head displacement), BCF (beat cross frequency), STR (straightness), LIN (linearity). Subsequently, the sperm concentrations were assessed with a microscopy-based hemocytometer.

### 4.4. Histology Examination

The histology staining was conducted according to the standard protocols. Briefly, testicular tissue from mice was fixed in 4% paraformaldehyde overnight on a room temperature shaker and routinely paraffin-embedded before sectioning. The paraffin-embedded testicular tissue was sliced into 5 μm thick sections and stained with hematoxylin–eosin (H&E) after being dewaxed and rehydrated. After staining, the sections were dehydrated and fixed in resin. Finally, they were observed using a light microscope (Nikon Ni-U, Tokyo, Japan).

Each tissue section underwent morphological analysis using a minimum of 6 randomly selected fields of view. The normal spermatogenesis tubules, tubular diameter, epithelium height, and lumen width were manually counted and measured by three individual investigators.

### 4.5. Untargeted Metabolomics Study

The sample preparation procedures were according to our previously reported protocols [67]. After thawing on ice, the samples were vortexed for 2 min until fully homogenized. Subsequently, 200 μL of each sample was mixed with 800 μL of methanol to eliminate proteins. Then, they underwent ultrasonic treatment in an ice water bath for 15 min, and the mixture was centrifuged at 4 °C and 14,500× *g* rpm for 15 min. The supernatant (800 μL) was then transferred to a fresh tube and dried in a vacuum concentrator at 37 °C, followed by reconstitution in 400 μL of methanol–water (1:1, *v*/*v*) by sonication on ice for 10 min. Upon completion, the mixture was centrifuged at 12,000× *g* rpm and 4 °C for 15 min. The supernatant was then transferred to a fresh glass vial for UPLC-Orbitrap-MS/MS analysis.

The metabolomic experiments were performed by NovoGene (Beijing, China) using a Thermo Fisher Scientific UPLC system (Dionex UltiMate 3000), and the Xcalibur software (version 3.0) was used for instrument control, data acquisition, and data analysis. The analysis method was according to that in previous studies [68,69]. The raw data were processed by Compound Discoverer 2.1 software (Thermo Fisher Scientific, Waltham, MA, USA), and identified metabolites using the mzCloud and mzVault libraries. MetaboAnalyst 5.0 (https://www.metaboanalyst.ca, accessed on 31 July 2023) was used to perform the principal component analysis (PCA) and partial least squares discriminant analysis (PLS-DA). Furthermore, the fold changes (FCs) of metabolites were calculated based on the changes in peak area average levels, and *p*-value was computed using an unpaired Student’s *t*-test. The metabolites with FC > 2 and *p* < 0.05 were deemed as the differential metabolites, and the KEGG database was applied to functionally annotate these differential metabolites.

### 4.6. Quantitative Real-Time Polymerase Chain Reaction (PCR)

Each sample’s total RNA was extracted using Trizol reagent (Invitrigen, Carlsbad, CA, USA). A Color Reverse Transcription Kit (EZBioscience, Suzhou, China) was used to reverse-transcribe the RNA to cDNA. The 2× Color SYBR Green qPCR Master Mix (EZBioscience, Suzhou, China) was used for real-time PCR, performed on a Quant Studio 6 Real-Time PCR System (Thermo Fisher Scientific, USA). Gene expression levels were determined using the efficiency-corrected 2^−ΔΔCT^ method, with β-actin serving as the internal control. Data were presented as fold change relative to the control groups. The primer sequences utilized in this study are provided in Supplementary Table S1.

### 4.7. Statistical Analysis

Data analysis and visualization were conducted using GraphPad Prism 6.0 and SPSS Statistics 26 software. The results are presented as means ± SEM. Group differences were assessed using two-sided unpaired Student’s *t*-test (for two groups) or one-way ANOVA test (for multiple groups). Spearman’s correlations were computed using the R software (v3.6.3) and visualized using the OmicStudio tools (available at https://www.omicstudio.cn). A significance level of *p* < 0.05 indicated statistical significance, while *p* < 0.1 represented a trending difference.

## 5. Conclusions

In conclusion, the findings of this study demonstrated that busulfan causes abnormal spermatogenesis and decreased sperm quality. The room-temperature WLZ extracts showed greater therapeutic potential in busulfan-induced male testicular damage compared to heated extracts. In the in vivo model, the alleviating effects of two WLZ room-temperature extracts on busulfan-induced testicular damage were also confirmed. The underlying mechanism may be attributed to the regulation of oxidative stress (Gpx1) and inflammatory factors (IL-1β, TNF-α, and IL-6) through arginine biosynthesis. From an application perspective, this study provides a new and promising direction for the treatment of busulfan-induced adverse effects.

## Data Availability

The raw data supporting the conclusions of this article will be made available by the authors on request.

## Supplementary Materials

The following supporting information can be downloaded at: https://www.mdpi.com/article/10.3390/ijms25126320/s1.
