# Supplementary material for: Arginine Biosynthesis Mediates Wulingzhi Extract Resistance to Busulfan-Induced Male Reproductive Toxicity
Source: Int J Mol Sci. 2024 Jun 7;25(12):6320. doi: 10.3390/ijms25126320 (PMC11203605; doi:10.3390/ijms25126320)
Supplement: Supplementary file 1 [file ijms-25-06320-s001.zip › ijms-3025423-supplementary.pdf]

Supplementary Material

Arginine Biosynthesis Mediates Wulingzhi Extract Resistance to Busulfan-Induced Male Reproductive Toxicity

Zifang Wu [1,†], Yuxuan Ma [1,†], Shaoxian Chen [1], Yuyan Liu [1], Xianglin Liu [2], Heran Cao [1], Tianqi Jin [1], Long Li [1], Mengqi Huang [1], Fangxia Yang [2,*] and Wuzi Dong [1,2,*]

[1] College of Animal Science and Technology, Northwest A&F University, Xianyang 712100, China; zifang_wu@126.com (Z.W.); mayuxuanrl@126.com (Y.M.); 15717875330@163.com (S.C.); 13683402668@163.com (Y.L.); caoheran@nwsuaf.edu.cn (H.C.); jintianqi2000@126.com (T.J.); 2021060188@nwafu.edu.cn (L.L.); h2036837547@163.com (M.H.)

[2] College of Forestry, Northwest A&F University, Xianyang 712100, China; lxl6260686@163.com

* Correspondence: yangfangxia@nwsuaf.edu.cn (F.Y.); dongwuzi@nwsuaf.edu.cn (W.D.)

† These authors contributed equally to this work.

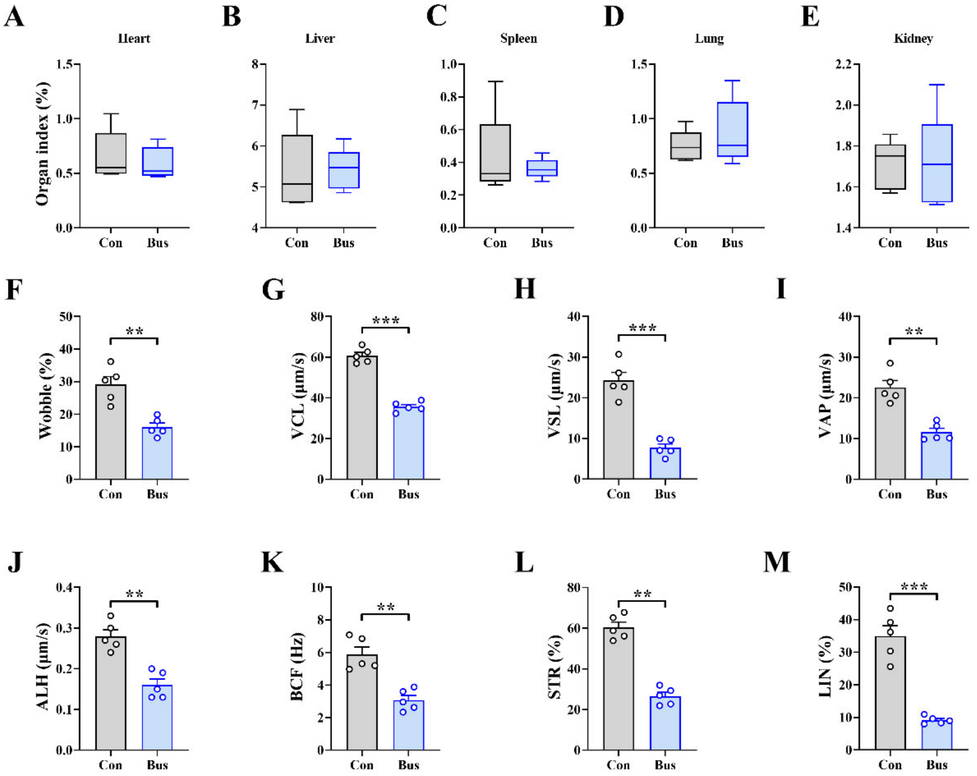

Supplementary Figure S1. Effect of busulfan on organ index and sperm parameters of mice. (**A-E**) The organ index (tissue weight/body weight × 100%) of heart (**A**), liver (**B**), spleen (**C**), lung (**D**), and kidney (**E**). (**F-M**) Sperm parameters was assessed by computer-aided sperm analysis; (**F**) wobble; (**G**) VCL, curvilinear velocity; (**H**) VSL, straight-line velocity; (**I**) VAP, average path velocity; (**J**) ALH, amplitude of lateral head displacement; (**K**) BCF, beat cross frequency; (**L**) STR, straightness; (**M**) LIN, linearity. n=6 for each group. All data are presented as means ± SEM. Statistical significance was determined by unpaired Student's t-test. $^{**}P < 0.01$, $^{***}P < 0.001$.

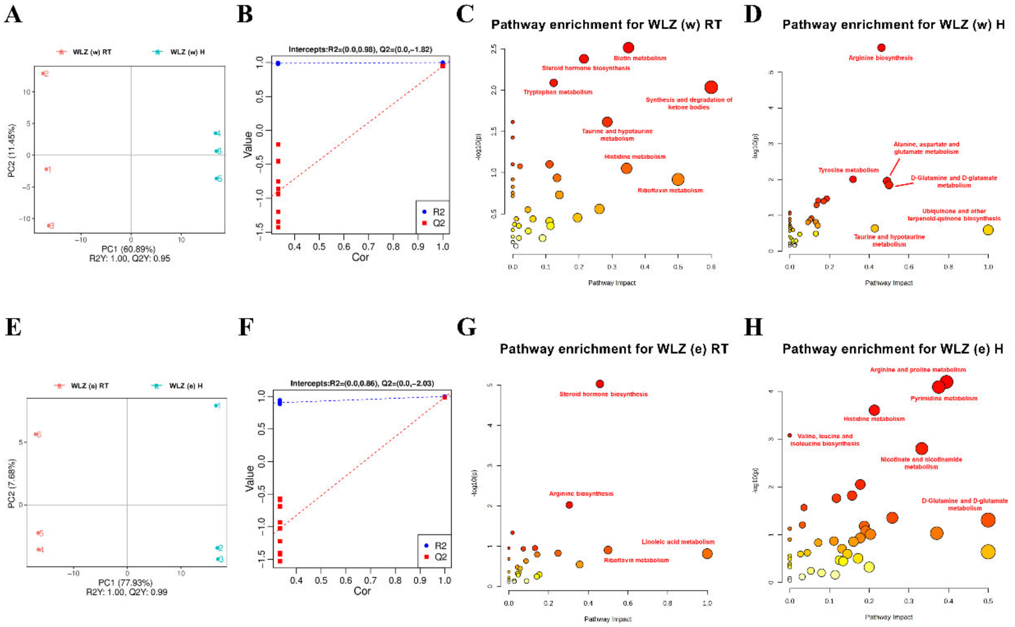

Supplementary Figure S2. The metabolome changes of WLZ water extract and WLZ ethanol extract at different temperature. (**A-D**) The metabolomics changes of WLZ (w) RT and WLZ (w) H; (**A**) the Partial Least-Squares-Discriminant Analysis (PLS-DA) score plot; (**B**) permutation test; (**C-D**) the pathway enrichment analysis of differential metabolites in WLZ (w) RT and WLZ (w) H. The darker the color and larger the shape of the circle, the stronger the pathway influence. (**E-H**) The metabolomics changes of WLZ (e) RT and WLZ (e) H; (**E**) the Partial Least-Squares-Discriminant Analysis (PLS-DA) score plot; (**F**) permutation test; (**G-H**) the pathway enrichment analysis of differential metabolites in WLZ (e) RT and WLZ (e) H. The darker the color and larger the shape of the circle, the stronger the pathway influence. n=3 for each group.

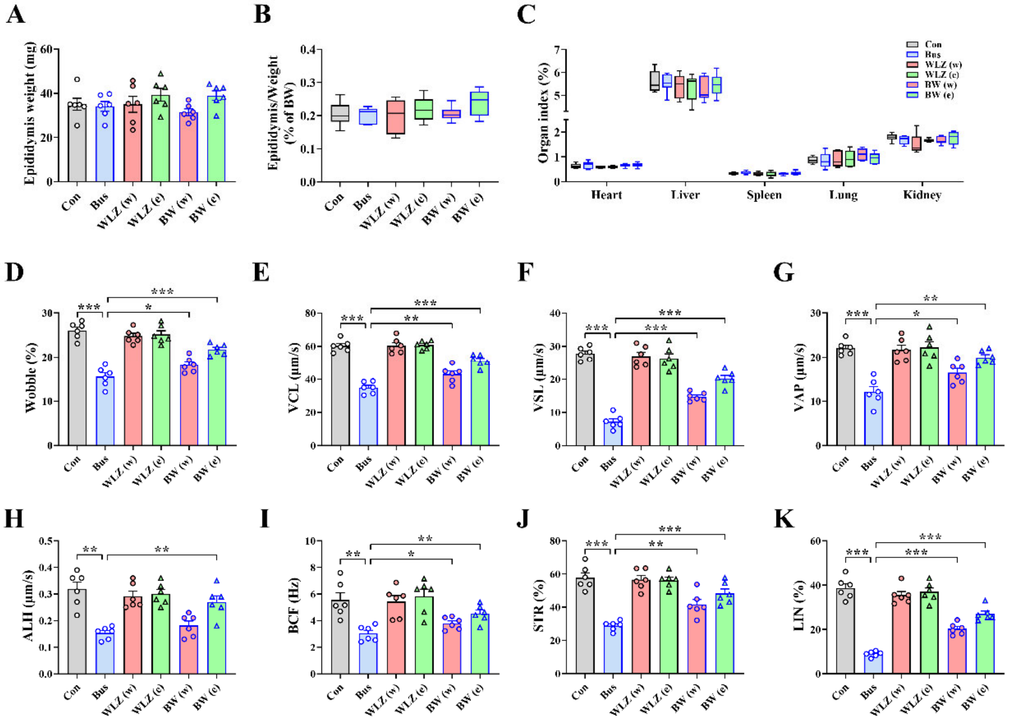

Supplementary Figure S3. Effects of different WLZ extracts on testis injury caused by busulfan. (**A**) Average epididymis weight. (**B**) Ratio of epididymis weight/body weight. (**C**) The organ index (tissue weight/body weight × 100%) of mice. (**D-K**) Sperm parameters was assessed by computer-aided sperm analysis; (**D**) wobble; (**E**) VCL, curvilinear velocity; (**F**) VSL, straight-line velocity; (**G**) VAP, average path velocity; (**H**) ALH, amplitude of lateral head displacement; (**I**) BCF, beat cross frequency; (**J**) STR, straightness; (**K**) LIN, linearity. n=6 for each group. All data are presented as means ± SEM. Statistical analysis was done using one-way ANOVA followed by Sidak's multiple comparisons test. *$P < 0.05$, **$P < 0.01$, ***$P < 0.001$.

Table S1. Primer sequence for qPCR in this study.

Gene	Sequence (5’-3’)	
β-actin	Forward:	GGTCATCACTATTGGCAACGAG
	Reverse:	GAGGTCTTTACGGATGTCAACG
Brdt	Forward:	AGGGAAGCCAGTGAAAGCAT
	Reverse:	TCCTTCGCATTATGCTCCAG
Tdrd7	Forward:	CTAAGGGCTGTCCTGCAGTC
	Reverse:	TGAGAGTTGCCTTTGGCTTT
Adam3	Forward:	GGTAACGACAGCCAGCAGTAAT
	Reverse:	GCTTCTTGGTTGTGGTCTTCTT
TNP2	Forward:	GTAGCTCAGGGCGAAGATACAA
	Reverse:	TTCCTGTGACATCATCCCAAC
Spata19	Forward:	TGTCCAAAGAGTGTCCACCTCA
	Reverse:	CAAGGGCTCAGCGTTTAGAGT
NANOS2	Forward:	GGTTGTGTGTCCCATCCTGAGG
	Reverse:	AGAGACTGCTGACTGCTGTTGAG
PLZF	Forward:	GACCTGGATGACCTGCTGTATGC
	Reverse:	CGAGCCTTACGGTCCTCTTCTTC
Kit	Forward:	CTCTGCGTCCTGTTGGTCCTG
	Reverse:	ACTCTGATTGTGCTGGATGGATGG
DAZL	Forward:	TGTGGACCGAAGCATACAGACAG
	Reverse:	TAAGCACTGCCCGACTTCTTCTG
Sohlh1	Forward:	GAGCGGGCCAATGAGGATTA
	Reverse:	TCTCTCGCTGACCACGTTTC
Smc3	Forward:	TGGCGGGCAACAGTGAACAG
	Reverse:	ACCTCGCAATTCCTCGCTCTTC
SYCP3	Forward:	GAGCCGCTGAGCAAACATCTAAAG
	Reverse:	AAAGGTGGCTTCCCAGATTTCCC

TNP1	Forward:	ATGGCATGAGGAGAGGCAAGAAC
	Reverse:	TCGCCCCGTTTCCTACTTTTCAG
Acrv1	Forward:	GAATCAAGTGAGCATGCTGTAG
	Reverse:	CTGAAGTGTGTTCTGACAAGTG
Lzumo3	Forward:	TCTCACATCGGAGGAAAATGAA
	Reverse:	CCAGTTTGCATTTCTGACTCAG
NAGS	Forward:	CTTCAAGCACAGTGACGGTAGC
	Reverse:	AGTTCGTAGGAGTCTCGGATGTC
OTC	Forward:	CCCAGAGGCAGAGAATAGAAAGTG
	Reverse:	ATAAGAAGAGTGATCCAAGGCAAGG
ASS1	Forward:	GTGGCTGAAGGAACAAGGCTATG
	Reverse:	TTGCTCACATCCTCAATGAACACC
ASL	Forward:	TTGGACAAGGTTGCTGAAGAGTG
	Reverse:	GCTCGTTGGCTGTGTGGATG
Nrf2	Forward:	GTTGCCACCGCCAGGACTAC
	Reverse:	GTGCTCAGAAACCTCCTTCCAAAAC
SOD	Forward:	TGTTACAACTCAGGTCGCTCTTCAG
	Reverse:	CTTGATAGCCTCCAGCAACTCTCC
CAT	Forward:	ATAGCCAGAAGAGAAACCCACAGAC
	Reverse:	TCCCTCGGTCACTGAACAAGAAAG
Gpx1	Forward:	GCAATCAGTTCGGACACCAGAATG
	Reverse:	CTTCTCACCATTCACTTCGCACTTC
Sirt1	Forward:	CGTCTTGTCCTCTAGTTCCTGTGG
	Reverse:	CCTCTCCGTATCATCTTCCAAGCC
IL-1β	Forward:	GCTGCTTCCAAACCTTTGAC
	Reverse:	AGCTTCTCCACAGCCACAAT
TNF-α	Forward:	ACGCTCTTCTGTCTACTGAACTTCG
	Reverse:	TGGTTTGTGAGTGTGAGGGTCTG

IL-6	Forward:	TTCTTGGGACTGATGCTGGTGAC
	Reverse:	GTGGTATCCTCTGTGAAGTCTCCTC
IFN-γ	Forward:	TGGAGGAACTGGCAAAAGGATGG
	Reverse:	ATGACGCTTATGTTGTTGCTGATGG
